# Developing online communication training to request donation for vascularized composite allotransplantation (VCA): improving performance to match new US organ donation targets

**DOI:** 10.1186/s12909-024-05026-9

**Published:** 2024-01-22

**Authors:** Laura A. Siminoff, Gerard P. Alolod, Hayley McGregor, Richard D. Hasz, Patricia A. Mulvania, Laura K. Barker, Heather M. Gardiner

**Affiliations:** 1 Department of Social and Behavioral Sciences College of Public Health, Temple University, Philadelphia, PA USA; 2 Gift of Life Donor Program, Philadelphia, PA USA; 3 Gift of Life Institute, Philadelphia, PA USA

**Keywords:** Donation, Vascularized composite allotransplantation, Program development, Program evaluation, Education

## Abstract

**Background:**

Approaching families of dying or newly deceased patients to donate organs requires specialized knowledge and a mastery of relational communication. As the transplantation field has progressed, Donation Professionals (DPs) are also leading conversations with family decision makers (FDMs) about the donation of uncommon anatomical gifts, such as face, hands, genitalia, referred to as Vascularized Composite Allotransplants (VCA) without much training or experience. To address the need for training, we adapted and beta tested an evidenced-based communication training program for donation discussions to VCA requests. The overarching goal of *C*ommunicating *E*ffectively *a*bout *D*onation for *V*ascularized *C*omposite *A*llotransplantation (CEaD-VCA) is to increase the number of VCA authorizations and to improve the socioemotional outcomes of FDMs.

**Methods:**

We developed CEaD-VCA, an online, on-demand training program based on the previously tested, evidenced-based communication skills training program designed to train DPs to have conversations about solid organ donation. The training was modified utilizing data from a national telephone survey with DPs and results of 6 focus groups conducted with members of the general public. The survey and focus groups assessed knowledge, attitudes, and barriers to VCA donation. The training was shaped by a partnership with a leading industry partner, the Gift of Life Institute.™

**Results:**

Using the results as a guide, the existing CEaD training program, consisting of interactive eLearning modules, was adapted to include technical information about VCA, foundational communication skills, and two interactive example VCA donation request scenarios to facilitate active learning. Forty-two DPs from two partner Organ Procurement Organizations (OPOs) participated in the beta test of CEaD-VCA. Pre- and post-test surveys assessed the impact of the training.

**Conclusions:**

The training was scored highly by DPs in effectiveness and ease of use. This project created a standardized, accessible, and comprehensive training for DPs to communicate about VCA donation. CEaD-VCA is an example of how to develop a communication skills training for difficult conversations utilizing input from stakeholders, guided by communication theory. It also demonstrates how gaps in communication skills during medical education can be filled utilizing advanced online Learning Management Systems. The training specifically addresses new CMS rules concerning OPO performance metrics.

## Background

### Supporting organ donation performance

Organ donation discussions asking families of dying or newly deceased patients to donate require a multitude of skills inclusive of technical expertise coupled with a mastery of relational communication. Most recently, the transplantation industry has been under intense criticism concerning performance and equity adequacy. The organ donation system is regulated by the federal government through Centers for and Medicare and Medicaid Serves (CMS) and Health Resource Service Administration (HRSA) who oversee policy the 56 federally funded Organ Procurement Organizations (OPOs) who are charged with recovering and working with United Network for Organ Sharing (UNOS) and hospitals to place and transport transplantable organs for transplantation.

The system of obtaining organs for transplantation is based in volunteerism in which individuals decide to donate the ‘gift of life.’ Americans have two main routes to donate: [1] pre-designate themselves as organ donors by registering as a donor on a state registry; 23% of individuals who are eligible to become an organ donor on death are registered [2]. The majority (77%) [1] of individuals who are eligible to donate will not have pre-registered and the decision as to whether to donate falls to their families during a time of crisis, when individuals are close to death or declared brain dead. The process of asking families of dying patients to consider the opportunity to donate organs requires a high level of skill [2–7] in the US, success in explaining the need for organ donation has been the subject of much research and even controversy. The practices of OPOs, especially as they relate to interacting with the families of minoritized communities, have been scrutinized in recent Congressional hearings and new rules to require a greater amount of accountability and higher levels of performance are being implemented by CMS [8, 9].

Adequate communication skills to expertly execute the technical and relational conversations that are required are required to meet new CMS requirements. These skills are not routinely or comprehensively part of medical/healthcare practitioner education [10, 11], and existing continuing education programs and interventions have been hard to scale, expensive, and time-consuming [11]. This lack of skills training is a lacuna in healthcare providers’ education and adversely impacts patients and families, as well as the providers themselves [12–16]. Practitioners have reported anxiety and struggles with their own emotions while holding these conversations [15]. This can result in provider delay or avoidance providing critical information to patients and families [17]. Moreover, when patients and families interact with clinicians with poor communication skills, they are more likely to perceive the patient’s care as poor [18], experience negative arousal [19], inhibit the grief process [18], and report post-traumatic stress [20].

Discussions with families to donate organs or tissues for transplantation to one of the over 100,000 patients on the national transplant waitlist and the millions in needs of human tissue [21] are challenging. The pronouncement of brain death and presentation of the option of solid organ and/or tissue donation involve several difficult end-of-life conversations, including discussions of the patient’s diagnosis, prognosis, and death determination [4, 22]. In some cases, additional gifts for research [23] are requested, including brain [24] or whole body donation [25]. Perhaps the most difficult donation conversation is the request for vascularized composite allografts for allotransplantation (VCA) [26].

### Vascularized composite allotransplantation

Recent advancements in transplant science makes it possible for multiple tissue types sharing a vascular structure (e.g., skin, muscle, bone, nerves, and blood vessels) to be transplanted from a donor to a patient. Patients who might benefit from a VCA allograft are individuals who have exhausted options for traditional reconstructive surgery or treatment to restore function for the face, larynx, upper and lower extremities, abdominal wall, and other structures such as a bladder, penis, and uterus. VCA transplantation can mean a tremendous increase in quality of life for these patients, by not only reducing pain but also restoring mobility and independent living skills, such as the ability to feed and dress themselves [27, 28]. The potentially positive impact of VCAs underscores the utmost importance of the conversation around VCA donation to patients awaiting restorative function and greater independence. In this paper, we describe the process and elements of teaching healthcare providers to hold difficult conversations through an evidence-based, online interactive skills training program focused on VCA donation.

## The context of VCA donation

Although the first successful VCA transplant occurred in 1998, the procedure is still relatively rare and continues to evolve [29, 30]. It was not until 2013 that VCA became classified as a type of organ donation; it had previously been listed as a type of tissue donation [31, 32]. Nonetheless, it remains fairly uncommon [33] and largely unknown to the public [34] as compared to typical solid organ donation, such as heart and kidney. To date, there have been 118 VCA transplants in the US [35], with 18 candidates listed on the national waitlist for VCA allografts as of September 13, 2022. VCA donors are also scarcer than solid organ donors. Only certain individuals can donate VCA allografts and, with the exception of uterus donation, VCA allograft donations can only be provided by deceased donors. There is currently no way to pre-designate one’s intent to be a VCA donor on donor registries. Thus, family decision makers (FDMs), who are usually the deceased’s legal next-of-kin, are tasked with the decision to authorize the donation of VCAs during already difficult circumstances.

Organ Procurement Organizations (OPOs) manage the authorization and recovery of VCA. Because the VCA donation opportunity is rare, OPO DPs must be able to deploy critical skills specific to these unique requests [36]. The lack of training or practice with these sensitive conversations and the unfamiliarity by the American public about VCA, make family discussions even more challenging when the opportunity for a VCA donation actually occurs. The vast majority of adults in the US have not heard of VCA donation, and nearly half say they would definitely refuse to donate their own or loved one’s tissues for VCA [37]. We also know of no evidence-based training programs for DPs about VCA, nor industry guidelines to manage these requests [38].

### The importance of communication skills for donation professionals

More than two decades of research with DPs and FDMs have demonstrated that high quality communication is key to informed decision-making, donor family satisfaction, and successfully obtaining family authorization to donation [2, 4–6, 39–41]. The communication skills required include both what is said (content) and how it is said (affect). The benefits of effective communication skills are positively and strongly associated with the donation decisions of FDMs, including longer and more detailed donation conversations, expressions of empathy and concern, creating positive connections with FDMs [42, 43] as well as validation of FDMs feelings and decisions [44]. In prior organ and tissue donation studies, several conversational topics were found to be important for successful request outcomes [45–47]. In general, it has been reported that DPs who are comfortable fostering positive, connective, and family-centered conversations are more successful in obtaining authorization [2, 7]. These skills are even more critical when a VCA donation discussion occurs. Given the unique and particularly challenging task of explaining VCA donation, DPs need robust communication skills to inform, educate, and engage in active dialogue, including a discussion of the benefits of VCA. These discussions need to be persuasive but not coercive [7].

### Teaching healthcare professionals to hold difficult conversations

There is no uniform training on how to hold difficult end-of-life conversations, and advanced communication or empathy training is not the norm for future clinicians [10]. Despite attempts to create continuing education programs to teach providers how to lead these conversations, most are conducted in person and often require expensive simulated patients [13, 48–50]. For DPs throughout the US, standardization is lacking across OPOs with most training occurring inhouse or relying on an outdated ‘see one, do one, teach one’ approach. Training programs offered by consulting firms and OPO-based institutes are also generally inperson and difficult to coordinate with DPs’ hectic schedules. In addition, available programs can be impractical, requiring concentrated time out of the field and sometimes costly travel, and most are not evidence-based, reinforced, or easily replicable. Nationwide, most OPO-provided trainings do not regularly and systematically address communication skills [51]. As such, the inconsistent, impractical, and inadequate preparation of DPs has been shown to negatively impact family discussions about organ and tissue donation [39, 52].

## Methods

### CEaD program development

The Communicating Effectively about Donation program (CEaD) was created to fill the need for a comprehensive, online communication skills training for DPs. First developed over 25 years ago to support teaching DPs informed decision-making and communication skill acquisition, Siminoff and colleagues have explored the various aspects of the organ and tissue donation process to inform the continuous development and refinement of the CEaD protocol [7, 44, 53]. The CEaD intervention trains DPs to have effective discussions with donor-eligible patients’ families through introducing, teaching, and practicing key relational and instrumental communication skills [54–57]. The CEaD program is based on evidence gathered in more than 25 years of data about the organ and tissue donation context [7, 44, 53, 58]. It was orginially developed to train DPs to discuss solid organ donation using simulated patients [57], but it has been adapted for completely online delivery [56, 57]. The theoretical foundation of CEaD interventions is Anderson’s theory of adult communication skill acquisition [59]. The model describes three stages of skill acquisition including the declarative stage where information and instruction are shared, the knowledge compilation stage where information is applied to the appropriate context, and the procedural stage where it is refined through repetition and feedback. It is also informed by Activity Theory of technology-mediated learning [60, 61]. Evaluations of the CEaD program showed that it improved DPs’ relational communication skills and the quality of the communication with all families; it also increased FDMs donation rates in some populations [56]. The CEaD program is demonstrated as most effective with novice requesters [56], and was successful in increasing DPs’ comfort levels discussing donation and the breadth of topics they discuss [55]. Delivery and implementation of the CEaD program has been accomplished through the RE-AIM framework which measures the real world impact of healthcare interventions and their sustainability from an individual and organizational prospective [62].

### CEaD for vascularized composite allotransplantation

The CEaD-VCA was adapted to specifically train DPs to discuss and obtain authorization for Vascularized Composite Allotransplantation as a web-based eLearning program [36, 63]. This developmental study was informed by data collected from the general public and from OPOs to understand DPs’ educational and communication needs to discuss VCA donation with FDMs and to obtain an understanding of the informational and attitudinal barriers present within the public. Focus groups with members of the general public identified strong initial reactions to VCA and little prior knowledge of the procedure [34]. Participants expressed concerns about mutilation of the patient and transference of identity, or facial recognition of the donor. Given the current infrequency of VCA transplantation have occurred in the US, it is not surprising that our results found that 70% of DPs have never had a conversation with an FDM about VCA and only 44% had had any training related to VCA [36]. Moreover, three-quarters of DPs who reported having prior training said it was inadequate. More than 7 in ten DPs reported that they would like to receive comprehensive training on VCA, and a majority requested that the training cover technical aspects of VCA donation and VCA communication approaches [36].

### E-Learning program development

The CEaD-VCA training was developed using Moodle, a learning management system (LMS) designed specifically to facilitate online teaching and learning. Moodle provides a variety of helpful features for users and administrators; it handles users’ access to the multiple didactic components of the training, records user activity, and ensures proper progression through the training by allowing administrators to restrict access to future modules until successful completion of preceding training components. To encourage active participation, the LMS can also restrict users’ ability to fast forward though educational media, such as video. Additionally, Moodle facilitates user registration by allowing trainees to create their own user accounts and log-in credentials. A required pre-training survey is administered as a part of the registration process, which must be completed before accessing the training. Upon successful registration, users receive an automated email containing their username, and a link to login to the training. The training can also be accessed through the CEaD-VCA homepage. Moodle also enables administrators to track user progress and provides access to metadata, such as the usage and the amount of time that it takes trainees to complete different components of the training. Usage data can be downloaded in CSV format for further analyses. Finally, the LMS allows trainees to save their work, complete the training in multiple sittings, and return to previously completed sections.

### Program functionality and user experience

**Fig. 1:**
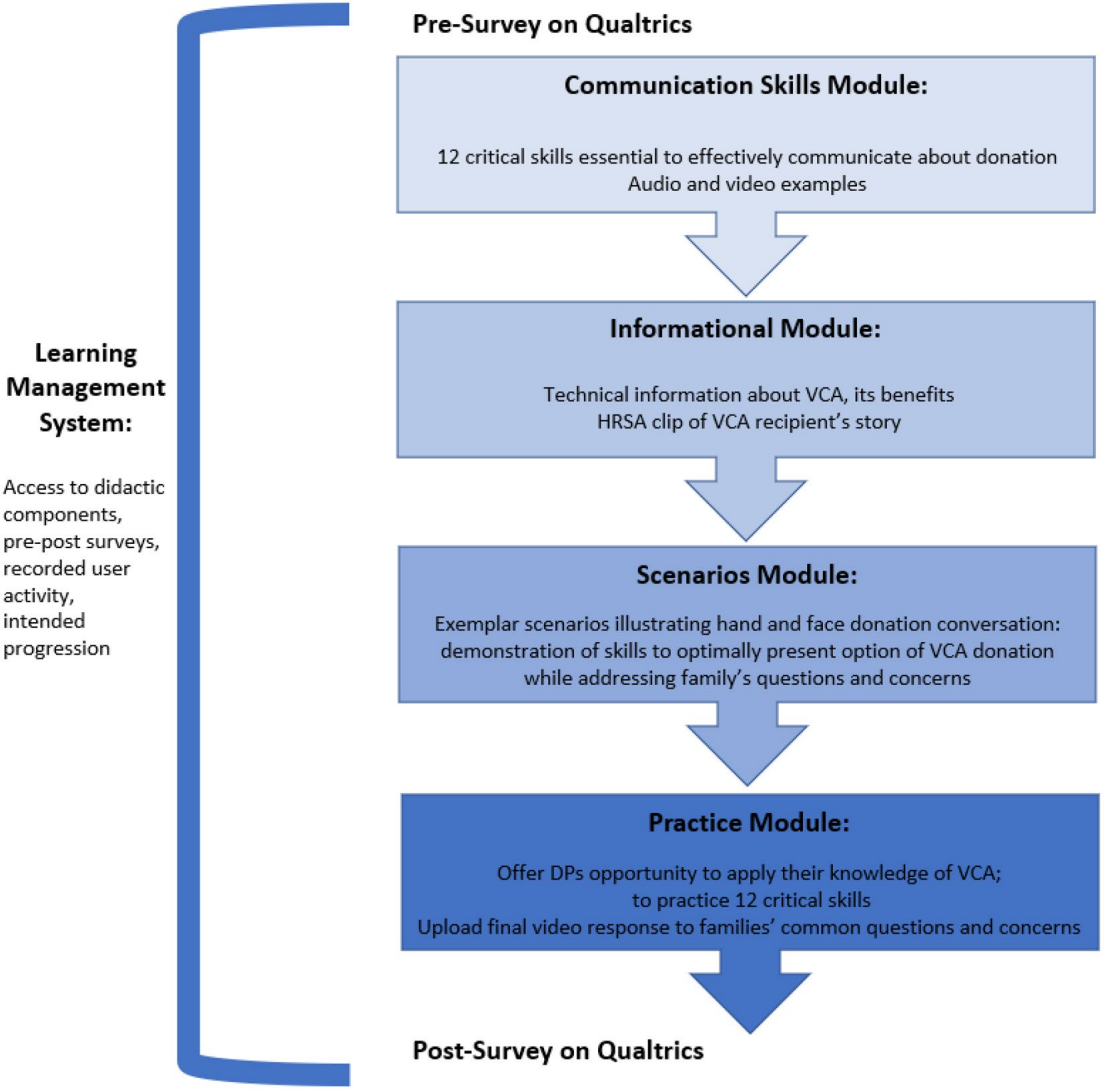
Description of the learning management system for CEaD-VCA

A learning path was determined for the CEaD-VCA training to ensure trainees completed all training components and progressed properly. The learning path consists of a landing page that serves as a homepage for trainees and contains general information about the training and its authors, a contact form, a training tutorial, a list of frequently asked questions, a troubleshooting guide, and access to a registration form and log-in portal. After logging into the training, trainees are presented with a brief introduction in addition to an overview of what their participation in the CEaD-VCA training will entail (Fig. 1).

Trainees begin with a Communication Skills Module. This module teaches 12 essential skills to communicate effectively about donation: empathy, offer of service, credibility, reassurance, repetition, legitimization, esteem, foot in the door, altruism, countering, apology, and related personal anecdotes. Trainees are provided with the definition and directives on when and how to appropriately use these communication skills. The purpose of this module is to increase the quality of the DPs communication with FDM; effective communication skills can build rapport, reduce uncertainty, and aid in the exchange of information [15, 64]. Additionally, audio and video examples of each skill are presented throughout the module to provide learners with sample language. Overall, the purpose of this first module is to reinforce how DPs should build rapport, reduce uncertainty, and exchange information with families in a way that is easily understood.

Next, trainees proceed to an Informational Module in which VCA is introduced through two videos. The purpose of this module is to establish a salient and tangible knowledge base about VCA for trainees. The first video lasts three minutes and delivers a technical definition of VCA, provides examples of VCAs, and describes the needs of potential recipients. Produced by Health Resources and Services Administration (HRSA), a second three-minute video provides a firsthand account of a combat veteran’s experience as a double arm transplant recipient.

The Scenarios Module demonstrates how to effectively discuss VCA donation with a family by utilizing the communication skills presented earlier in the training and demonstrating exemplary responses to commonly asked questions and concerns pertaining to VCA donation. Two scenarios illustrate in-person discussions about vascularized composite allograft donation to model interactions between DPs and FDMs. This module is guided by Activity Theory as knowledge acquisition from technology-mediated learning relies on engagement in meaningful, contextually appropriate activities [60, 61]. When knew information is both presented and enacted by the learner, associations between existing knowledge and new information are made, reinforcing new skills, and increasing enjoyment of the educational experience [65, 66].

Learners are able to practice their skills through two interactive scenarios: a conversation about face donation and one about hand donation. The scenarios branch at critical moments of the donation discussion to allow trainees the opportunity to take what they have learned in previous modules and apply their knowledge of VCA and the 12 communication skills. In this Practice Module, learners videorecord a response to FDM questions and concerns about face and hand donation and receive personalized feedback in the form of an individualized report card (Fig. 1).

Finally, after completion of the final skills practice, trainees are presented with a brief conclusion summarizing the training followed by a post-training survey. The DPs were asked to rate the useability of the training and rated it highly. The detailed results of the pre-and post- surveys have been published elsewhere, and covered each of the modules, content and usability [63]. A certificate of completion is available for their records.

### Creating the content of the training

The current version of the Communication Skills Module was adapted to comply with the CEaD-VCA LMS. In addition to aesthetic modifications, some of the language in the module was modified to reflect current terminology utilized within the field of organ and tissue donation (e.g., changing donation requestor to donation professional). The content and video examples contained in the CEaD-VCA Communication Skills Module are consistent with what was contained within the original CEaD training.

To create the three-minute descriptive introductory video about VCA, a storyboard outlining the different topics and visuals to be included in the final cut was constructed. Following the storyboard process, a script was developed for the purpose of narrating the video. A voiceover artist was hired to record the finalized script and a production company was hired to produce the video. The research team worked with the production company to identify stock videos and images available online to include in the video for the purpose of visual aid. Once all of the components were selected, the production company constructed the final product.

The content of the Scenarios Modules was developed by incorporating exploratory findings from the formative stages of the study [34, 36], in addition to collaboration with a clinical educator from the Gift of Life Institute. Storyboarding established scenes allowing for the accurate presentation of VCA-related information and demonstration of effective communication skills. A rigorous script-writing process ensured that scene dialogue, technical information, and the dramatized decision-making environments approximated reality and current practice. Trained professional actors were hired to portray FDM roles in a VCA donation discussion. A video production company with experience in creating scientific and educational content was hired to film, edit, and finalize the videos.

While the Scenarios Module illustrates DP-led conversations about face and hand donation, the Practice Module invites trainees to respond to 10 brief video clips (five face, five hand) of FDMs presenting common questions and concerns about face and hand donation. After viewing each clip, trainees record their video responses to the various prompts directly within the LMS interface. Trainees can record themselves as many times as they desired before ultimately submitting their response for evaluation by the research team.

To assess the functionality of the CEaD-VCA training before beta-testing, the team implemented an internal test of the program and its various components. Trial accounts were created for individual team members to access the training and to ensure that all the videos and LMS features, including the recording function within the Practice Module, worked properly. The pre- and post-training Qualtrics online surveys were also tested internally by the team to confirm that the skip logic and answer validation worked as intended. Minor changes to language were made based on the results from these preliminary tests.

As a secondary measure, the team partnered with LifeGift, a large OPO in Texas, to externally test the CEaD-VCA training program before it was pilot tested by two regional Organ Procurement Organizations (Gift of Life and LifeBanc). Ten DPs from LifeGift completed the CEaD-VCA training program in its entirety to confirm the efficacy of the registration process and to verify the flow and accessibility of the training modules. After completing the training, the team collected qualitative and quantitative feedback from DPs on the utility of the training, user experience, as well as the look and feel of the website. Following the beta-test, no changes were deemed necessary to the CEaD-VCA training.

## Discussion

The ongoing challenges of COVID-19 pandemic has brought to the forefront the need for communication skills instruction that is accessible, scalable, sustainable, and flexible while retaining the effectiveness of a typical didactic in-person training. These advantages, especially for those whose schedules are dictated by the demands of a clinical setting, makes e-learning advantageous for medical education and, especially, postgraduate and continuing studies. This study provides an example of how to take existing training and adapt it to provide asynchronous learning while maintaining an interactive component and providing learners with individualized feedback. The methods and LMS platform utilized effectively addressed new developments in medical technology while simultaneously updating the training to provide scalable, cost-effective training that was easy for health professionals to access and use.

Practitioners with high quality interpersonal communication skills are immensely valuable to institutions and critical to providing excellent care across the lifespan. However, most health systems fail to require demonstrable communication skills training and offer little relevant preparation. Much of the available training has been expensive and time-consuming, with in-person exercises that are simply out of reach to cash-strapped budgets and busy providers. However, new technologies provide opportunities for change. The CEaD-VCA is an example of the future of communication skills training programs using advanced LMS e-learning, evidence-based communication theory and stakeholder partnerships.

Innovations in LMS programming allow trainers to provide interactive content and skills practice in e-learning environments. The capabilities of LMS have dramatically advanced in the past few years [67]. Older, more static e-learning programs may find that they can increase their interactivity abilities to evaluate their program by re-tooling their programs using newer software applications. The LMS provided hands-on learning and opportunities for DPs to show (by recording and submitting videos) what they’ve learned both in general communication skills and about VCA donation. This modality for communication skills training provides medical schools, hospitals, and other entities the ability to address strategic gaps, like difficult end-of-life conversations. OPOs and hospitals often are aware of deficits in their providers communication skills but don’t have an efficient, systematic way to train individuals or evaluate application of the skills learned. Delivering the CEaD program exclusively using e-learning technology was based on the reality that DPs spend much of their time in hospital and have a much greater access to technology than before. Further, our extensive evaluation of the program using the RE-AIM framework demonstrated that an online-only version of the CEaD program could be highly effective for the VCA context [62].

## Conclusions

CEaD-VCA was successful because we engaged in significant formative and evaluative research protocols to ensure it was meeting the needs of stakeholders, e.g., OPOs and their DP staff. We engaged those partners during all aspects of the development process, creating and implementing feedback loops from the individuals who approach families about VCA, which itself is still an emerging subfield in the transplantation landscape. We did this while keeping communication theory and evidence-based communication research as the driving force of the training. This approach to training has only increased in its value to the field. New CMS regulations now require the highest level of productivity and improvement in performance of all OPOs [68]. The most effective way to increase the number of available organs is to increase the number of authorized patient donors.

Future iterations of CEAD-VCA could delve into implementation, noting the impact of the training on actual donations. Further work could be done to provide training specifically tailored for diverse FDM and could identify key ethical concerns related to VCA donation. As technological advances move a greater portion of providers’ professional life into the digital world and e-learning programs in adult education become more ubiquitous, next steps are to make greater use of Artificial Intelligence and chatbots to engage learners providing a real time “back and forth” level of interaction previously not available to asynchronous instruction [67].

## Data Availability

Data sharing is not applicable to this article as no datasets were generated or analyzed during the current study. Data from parent study can be requested from the corresponding author.

## Abbreviations

DPs: Donation professionals
FDMs: Family decision makers
VCA: Vascularized composite allotransplantation
CEaD: Communicating effectively about donation
OPO: Organ procurement organization
LMS: Learning management system
